# Heteroexpression of *Osa*-*miR319b* improved switchgrass biomass yield and feedstock quality by repression of *PvPCF5*

**DOI:** 10.1186/s13068-020-01693-0

**Published:** 2020-03-19

**Authors:** Yanrong Liu, Jianping Yan, Kexin Wang, Dayong Li, Yejun Han, Wanjun Zhang

**Affiliations:** 1 College of Biological Science, China Agricultural University, Beijing, 100193 People’s Republic of China; 2 College of Grassland Science and Technology, China Agricultural University, Beijing, 100193 People’s Republic of China; 3 National Energy R &D Center for Biomass (NECB), China Agricultural University, Beijing, 100193 People’s Republic of China; 4 Beijing Vegetable Research Center (BVRC), Beijing Academy of Agricultural and Forestry Sciences, National Engineering Research Center for Vegetables, Beijing, 100097 People’s Republic of China; 5 National Key Laboratory of Biochemical Engineering, Institute of Process Engineering, Chinese Academy of Sciences, Beijing, 100190 China

**Keywords:** Switchgrass, MiR319, *PvPCF5*, Lignin, Biomass yield

## Abstract

**Background:**

Switchgrass (*Panicum virgatum* L.), a C_4_ perennial grass, has been recognized as one of the most potentially important lignocellulose biofuel crops. MicroRNA319 (miR319) plays a key role in plant development, abiotic resistance, and cell wall biosynthesis by repressing expression of its target *TCP* genes. We hypothesized miR319–*TCP* pathway could play important roles in switchgrass feedstock characteristics for biofuel production, and produced switchgrass transgenic plants overexpressing miR319 (by ectopic expressing *Osa*-*MIR319b* gene), blocking miR319 (by overexpressing a target mimicry of miR319/*MIM319*) and repression of miR319 target gene *PvPCF5*. Plant phenotype, biomass yield, and feedstock quality of transgenic plants were analyzed.

**Results:**

Overexpression of miR319 in switchgrass promoted leaf elongation and expansion of transgenic plants, increased plant height, stem diameter, and resulted in a significant increase in plant biomass yield. Transgenic plants overexpressing of miR319 reduced lignin content, showed significantly higher enzymatic hydrolysis efficiency compared to the wild type plant. However, opposite results were observed in the *MIM319* plants. Furthermore, suppression of miR319 target gene *PvPCF5* activity also reduced lignin content, increased lignin monomer S/G ratio and the proportion of β-*O*-4 linkages, while significantly improving the sugar production per plant. Quantitative real-time (qRT-PCR) analysis indicated that expression of *PvMYB58/63B* and *PvHCT* with predicted TCP binding sites in their promoter regions was negatively regulated by miR319–*PvPCF5* module.

**Conclusions:**

MiR319–*PvPCF5* module plays positive roles in regulating biomass yield and quality of switchgrass. It can be utilized as a candidate molecular tool in regulating biomass yield and feedstock quality. The finding could also be transferred to other grasses for forage quality improvement through genetic manipulation.

## Background

To meet the energy crisis linked to the depletion of fossil fuel, biomass and biomass-derived biofuel as the renewable energy received recent attention [1]. The second-generation lignocellulosic bioenergy crops, wherein stems and leaves of plants, such as switchgrass [1], *Miscanthus* [2], and poplar [3], could be used for biofuel production to partially solve the energy challenge [4]. Among them, switchgrass (*Panicum virgatum* L.), a C_4_ perennial grass, has been recognized as one of the most potentially useful lignocellulosic biofuel crop [1].

However, lignocellulose materials are blocked by lignin in the secondary cell walls, resulting in a poor saccharification efficiency [5]. In recent decades, regulating the expression of lignin biosynthesis genes [6–10] or transcription factors (TFs) that involved in secondary cell wall regulation, including members of MYELOBLASTOSIS (MYB), NAM/ATAF/CUC (NAC), and APETALA2/Ethylene Responsive Factor (AP2/ERF) families [11–14], could efficiently alter lignin composition and structure and/or secondary cell wall remodeling, leading to enhanced sugar release efficiently [reviewed in 15, 16]. However, those improvements were also accompanied with potential side effects such as stunted plant growth [11–13], sterile characteristics [12], or sensitivity to biotic and/or abiotic stresses [17, 18]. Therefore, it is highly desirable to determine alternative methods of lignin engineering that can increase cell wall digestibility with a minimum impact on plant fitness and yield.

MicroRNA (miRNA), a kind of non-coding small RNA in plants, play key roles in plant development processes by repressing the expression level of its target genes [19]. In recent decades, several miRNAs and/or their target genes have been shown as potential molecular tools in improving biomass and/or saccharification efficiency in switchgrass by indirectly regulating plant development [20, 21]. MiR319, one of the most ancient miRNAs, was reported to post-transcriptionally regulate mRNA abundance of class II TCP (TEOSINTE BRANCHED1, CYCLODEA, PROLIFERATING FACTORS/PCF) transcription factors [22–26]. Higher miR319 level or lower *TCPs* content resulted in an excess of cell proliferation generation crinkled or wider leaves by regulating plant cell proliferation and elongation [22–26], delayed leaf senescence by repressing jasmonic acid (JA) biosynthesis [27], and affected flower development [28]. In *Arabidopsis*, the miR319–target class II TCPs, notably *AtTCP4*, have been reported to perform its biological function by activating cyclin-dependent kinase inhibitor 1 (ICK1) [29], DWARF4 (brassinolide (BR) biosynthesis key enzyme) [30] and LOX2 (lipoxygenase 2, a JA biosynthesis rate-limiting enzyme) [27]. BR and JA, two plant growth regulatory hormones, were reported highly correlated to plant biomass yield and lignin content [31, 32]. Furthermore, AtTCP4 directly activated *VASCULAR*-*related NAC*-*DOMAIN7* (*VND7*), a NAC subgroup gene, enhancing lignin and cellulose content and accelerating vessel formation [33]. In addition, overexpression of miR319 in switchgrass enhanced ethylene production and expression of down-stream genes of ethylene signaling transduction, such as upregulated expression of some ERFs [34]. Intriguingly, PvERF001 has been verified as a positively regulator in saccharification of switchgrass biomass [14]. These previous reports suggest miR319 and its target genes may play a key role in regulating biomass yield and secondary cell wall components in switchgrass.

To date, miR319–*TCP* module alterations to plant morphology focus mainly on leaf or flower [22–28], there are rare reports studying stem development characteristics, especially biofuel characteristics of stems. In this study, transgenic plants of overexpression of miR319 (OE-miR319), repression of miR319 (*MIM319*) and suppression of *PvPCF5* activity were used to investigate the roles that miR319–*PvPCF5* plays in regulating stem biomass yield and feedstock quality in switchgrass. Furthermore, we investigated the potential molecular mechanism of miR319–*PvPCF5* in negatively regulating lignin content by qRT-PCR. The results demonstrate miR319–*PvPCF5* pathway is of high potential in improving the biomass yield and feedstock quality of switchgrass, a C4 biomass plant.

## Results

### Expression profiles of miR319 and its target genes in switchgrass stems

To explore the function of miR319 and its target gene *PvTCPs* in switchgrass stem growth, development, and lignin deposition, we first tested the relative expression levels of miR319 and *PvTCPs* in four segments of the second internode from the top of the plant (Additional file 1: Figure S1a). The results showed the relative expression level of miR319 was significantly higher in the first segment (the lowest segment of internode), compared to the upper developed segments (6th, 12th and 18th) (Additional file 1: Figure S1b). Expression level of *PvPCF7* and *PvPCF8* was significantly higher in the upper segments (6th, 12th and 18th), compared to the first segment. However, expression of *PvPCF5* and *PvTCP21* was significantly higher in the first segment (Additional file 1: Figure S1c). As shown in Figure S1a, the upper segments of the internode were a darker brown color in phloroglucinol–HCl staining assay, indicating a higher lignin content. The results suggest that expression level of miR319 and its target genes have biological relevance to stem development and lignin deposition in switchgrass.

### Overexpression of miR319 improved switchgrass biomass yield

**Fig. 1:**
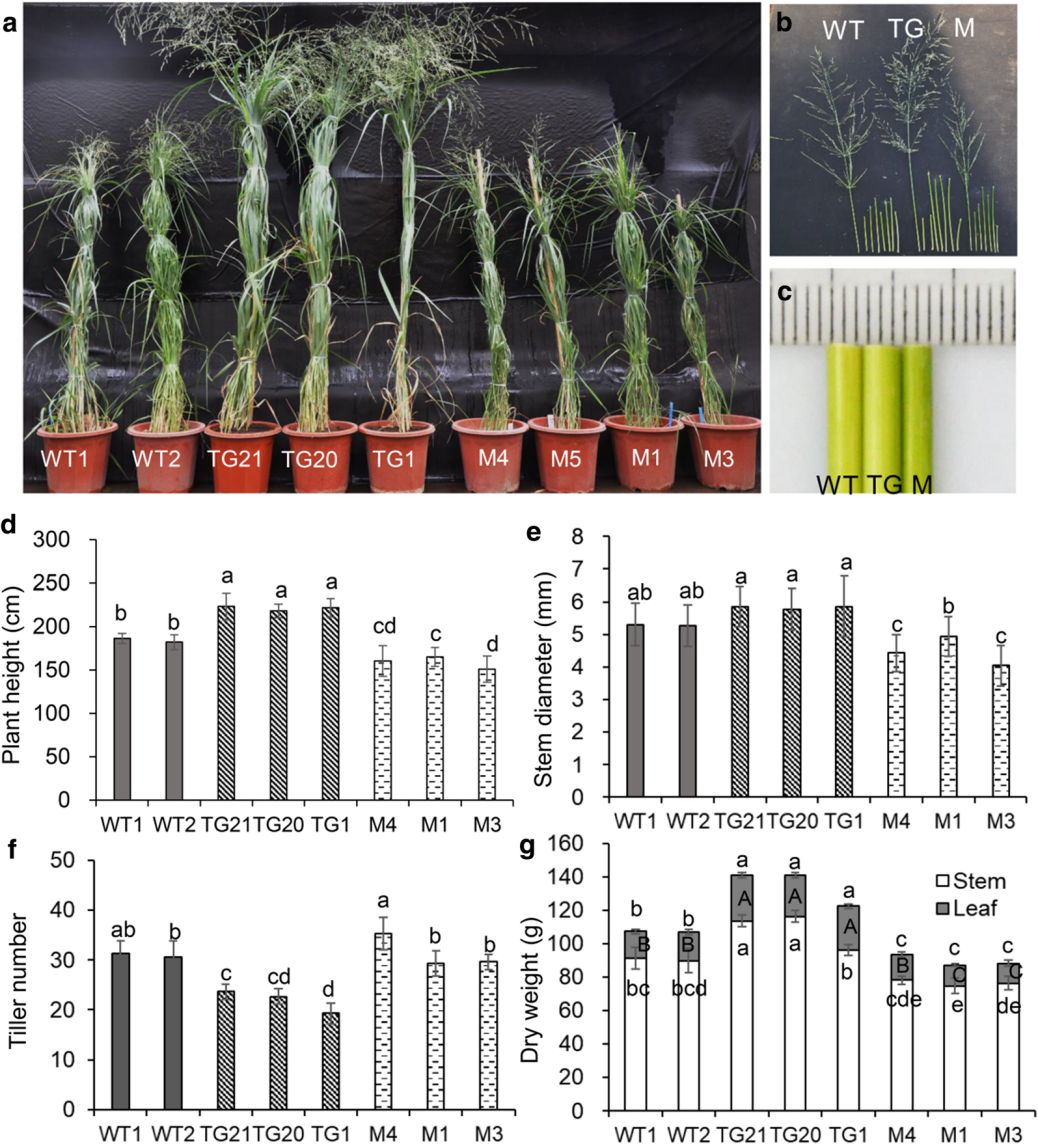
Modulated expression level of miR319 in switchgrass resulted in pleiotropic phenotype in transgenic plants. **a** Morphological characteristics of OE-miR319 (TGs) and *MIM319* (Ms) transgenic plants. **b** Stem internodes and inflorescence of WT and transgenic plants. **c** Comparison of the diameter of the bottom internode of WT and transgenic plants. **d** Analysis of plant height. **e** Analysis of first internode diameter. **f** Analysis of tiller number. **g** Analysis of aboveground dry biomass. The data are shown as the mean ± SD of four biological repetitions (**d**, **e** had 20 technical repeats; *N* = 4, *n* = 20). Different letters indicate statistically significant differences as determined by Duncan’s multiple range test (*P* < 0.05)

The switchgrass transgenic plants used in this study have been reported in our previous work, the expression of mature miR319 and its target genes in OE-miR319 plants had been analyzed in detail [34], and were briefly described (Additional file 2: Data S1). To investigate the function of miR319 in switchgrass stem development, we compared the phenotype of R3 stage tillers of 6-month-old OE-miR319 (TG) or *MIM319* (M) plants. The results showed that plant height of OE-miR319 was significantly higher (about 40 cm) than wild type (WT) plants (Fig. 1a, d), which was mainly due to the longer stem and inflorescence in OE-miR319 lines (Table 1; Fig. 1b). The *MIM319* lines were significantly shorter (about 20 cm) (Fig. 1a, d) than WT plants. Furthermore, while there was no significant difference in stem length between WT and *MIM319* plants, the length of the inflorescence axis of *MIM319* plants was only about half of that of WT plants (Fig. 1b; Table 1). Stem diameters of OE-miR319 plants were slightly thicker, but not significantly different from the WT. However, the stems of *MIM319* lines M3 and M4 plant were thinner than WT plant (Fig. 1c, e). Tiller numbers were significantly decreased in OE-miR319 plants compared to WT (Fig. 1f). A trend of slightly increased tiller numbers was observed in *MIM319* plants, but this was significant only in M4 line (Fig. 1f). Intriguingly, the weight of dry materials of stems (DMS) of OE-miR319 plants TG21 and TG20 was significantly greater than that of WT plant, DMS weight of TG1 was slightly greater, but not statistically different from WT. DMS weight of *MIM319* lines M1 and M3 was significantly less than that of WT plant (Fig. 1g). In comparison, OE-miR319 plants had longer, wider, and thicker leaves, while *MIM319* plants had shorter, narrower, and thinner leaves, compared to WT plants (Table 1). The results suggest expression level of miR319 positively affects switchgrass stem biomass yield.

**Table 1:** Characteristics of wild-type and transgenic plants

	Rachis length (cm)	Stem length (cm)	Leaf length (cm)	Blade width (mm)	Blade thickness (mm)
WT1	97.39 ± 10.68 bc	92.53 ± 3.46 de	65.78 ± 9.94 a	14.10 ± 1.97 b	0.15 ± 0.01 c
WT2	94.33 ± 5.56 c	89.91 ± 7.86 d	67.66 ± 10.69 a	14.46 ± 0.86 b	0.14 ± 0.01 dc
TG21	104.95 ± 10.51 ab	118.40 ± 14.56 a	62.85 ± 13.13 ab	17.04 ± 2.56 a	0.23 ± 0.03 a
TG20	104.62 ± 12.34 a	107.63 ± 7.61 bc	63.61 ± 10.00 a	17.30 ± 2.57 a	0.23 ± 0.03 ab
TG1	107.23 ± 10.5 ab	112.67 ± 5.65 ab	61.20 ± 7.68 ab	18.03 ± 2.27 a	0.21 ± 0.02 b
M4	59.79 ± 9.88 de	100.30 ± 6.90 cd	50.84 ± 4.29 c	9.95 ± 0.93 c	0.12 ± 0.01 de
M1	64.95 ± 5.98 d	100.74 ± 9.22 cd	55.94 ± 3.17 bc	11.14 ± 0.51 cd	0.11 ± 0.01 de
M3	54.17 ± 7.53 e	96.84 ± 16.31 de	49.46 ± 4.23 c	11.72 ± 1.09 d	0.11 ± 0.01 e

Each number is the mean of four biological repeats (with 20 technical repeats) ± SD. Different letters indicate statistically significant differences as determined by Duncan’s multiple range test (*P* < 0.05)

### Overexpression of miR319 reduced switchgrass lignin content

**Fig. 2:**
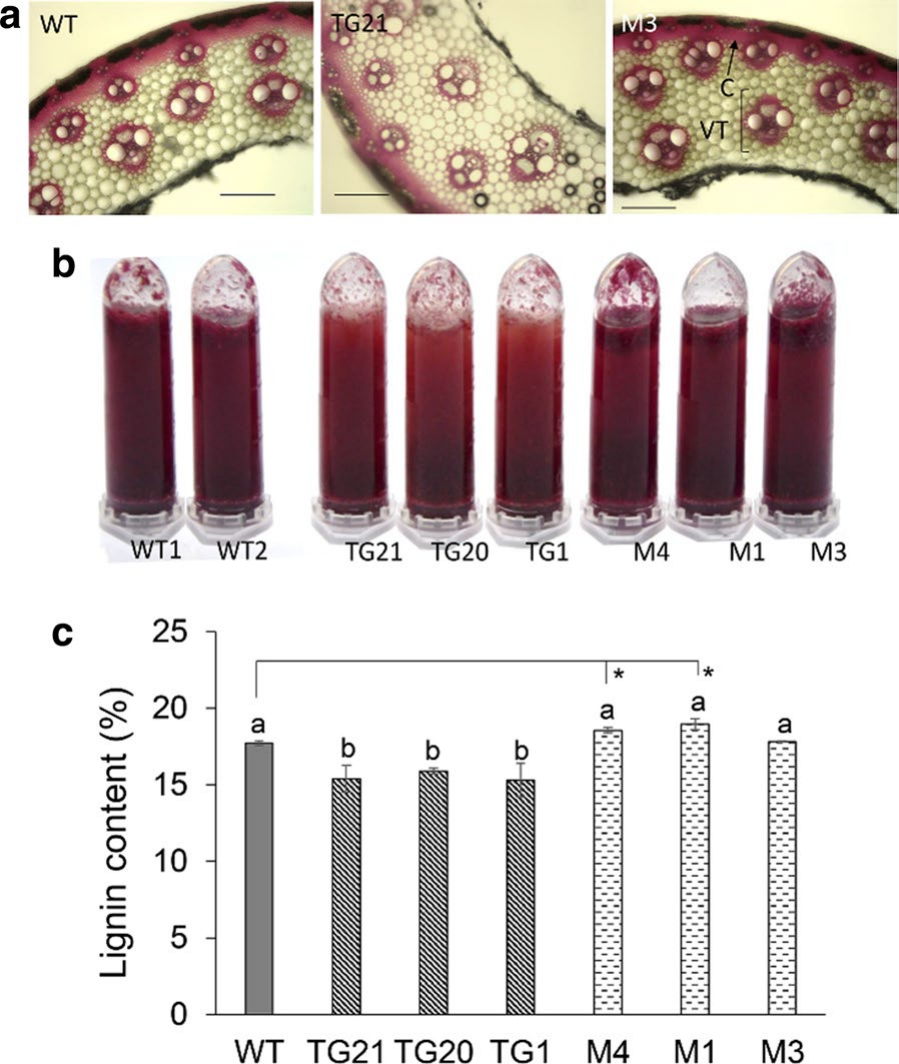
Analysis of lignin content in WT and miR319 transgenic plants. **a**, **b** Phloroglucinol–HCl staining assay of lignin content in the middle of the first internode of E3 stage cross-sections (**a**) and in the dry materials powder of stems **(b**). *C* collenchyma, *VT* vascular tissue. Scale bars indicate 5 μm. **c** The Klason lignin content of dry materials of stems. Data are shown as the mean ± SD (*N* = 3, *n* = 5). Different letters and asterisks represent significant differences compared to WT (*P* < 0.05)

We estimated lignin content of WT, OE-miR319, and *MIM319* plants by histochemical staining assay with phloroglucinol–HCl (a stronger red color indicates a higher lignin content). Hand-cut sections of the middle of the first internode from the top of the E3 stage tiller (1NE3) were stained with phloroglucinol–HCl. As shown in Fig. 2a, the red color stained mainly in vascular tissues (VT) and collenchyma (C), color depth was ranked as: TG21, WT, and M3. We also stained their DMS powder, OE-miR319 lines showed clearly lighter red color than WT and *MIM319* plant (Fig. 2b). We further measured the Klason lignin content of DMS of WT, OE-miR319, and *MIM319* plants. The results confirmed the lignin content of OE-miR319 plants was significantly lower compared to that of the WT (Fig. 2c). The lignin content of *MIM319* lines M1 and M4 was significantly higher than that of WT plants. Clearly, the results indicate the expression level of miR319 negatively affects the lignin content of switchgrass.

### Overexpression of miR319 improved sugar yield by enhancing enzymatic hydrolysis

**Fig. 3:**
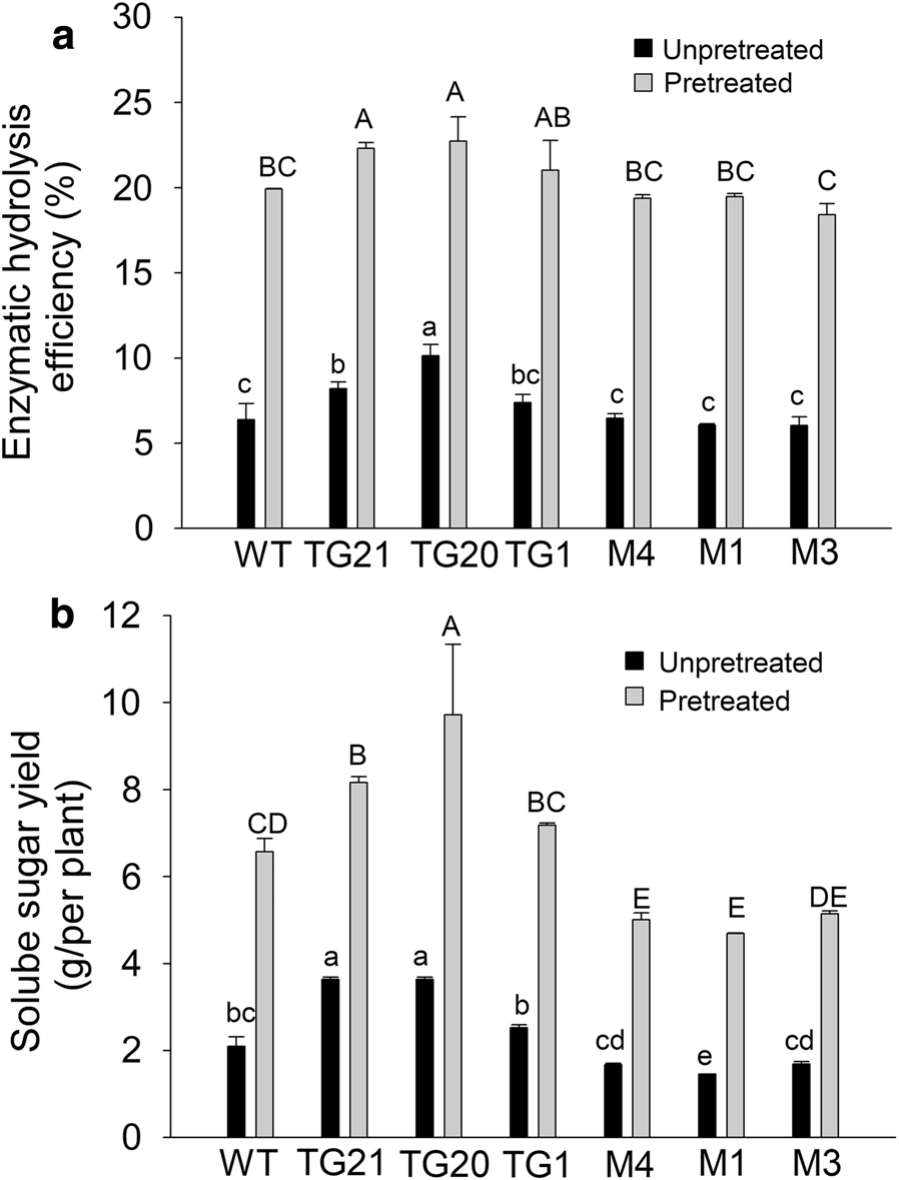
Effects of miR319 on switchgrass enzymatic hydrolysis efficiency and soluble sugar yield. **a** Comparison of enzymatic hydrolysis efficiency. **b** Soluble sugar yield tests. Data are shown as the mean ± SD (*N* = 3, *n* = 5). Different letters represent significant differences (*P* < 0.05)

Lignin is the major impediment to lignocellulose degrading [15, 16]. To analyze whether the change in lignin content of OE-miR319 and *MIM319* plants affected the enzymatic hydrolysis efficiency, we measured the enzymatic hydrolysis efficiency of cell wall residues (CWR) without pretreatment or after alkali pretreatment. There were no significant differences in cell wall carbohydrate compositions or total carbohydrate between WT and transgenic plants (Additional file 3: Table S1). Further study showed switchgrass is recalcitrant to saccharification, only about 6 percent of the total carbohydrate was released from CWR of WT plants after enzymatic hydrolysis (Fig. 3a). Without pretreatment, enzymatic hydrolysis efficiency of OE-miR319 line TG20 was the highest, and TG21 was significantly higher than that of WT. However, no significant differences were observed between *MIM319* lines and WT plants (Fig. 3a). After pretreatment with alkali, the enzymatic hydrolysis efficiency of all the tested lines was dramatically improved. Enzymatic hydrolysis efficiency of OE-miR319 plants was significantly increased to about 22.5%, which was significantly higher than that of WT and *MIM319* lines. The lowest enzymatic hydrolysis efficiency was observed in *MIM319* line M3 (Fig. 3a).

The calculation of soluble sugar yield per plant indicated that without pretreatment the total sugar yield of OE-miR319 lines TG21 and TG20 was significantly higher (3.6 g) than that of WT (2.1 g). After alkali pretreatment, sugar yield of TG21 and TG20 was increased to 9.7 and 8.2 g/plant, respectively, which was significantly higher than that of WT (6.6 g/plant) (Fig. 3b). The soluble sugar yield of *MIM319* lines (4.6–5.1 g/plant) was significantly lower than that of WT plants after alkali pretreatment. However, without pretreatment only M1 plants showed considerably lower soluble sugar yield than that of WT, soluble sugar yield of M4 and M3 were not significantly different from that of WT (Fig. 3b).

### Overexpression of *PvPCF5*-*SRDX* chimeric gene reduced switchgrass lignin content

**Fig. 4:**
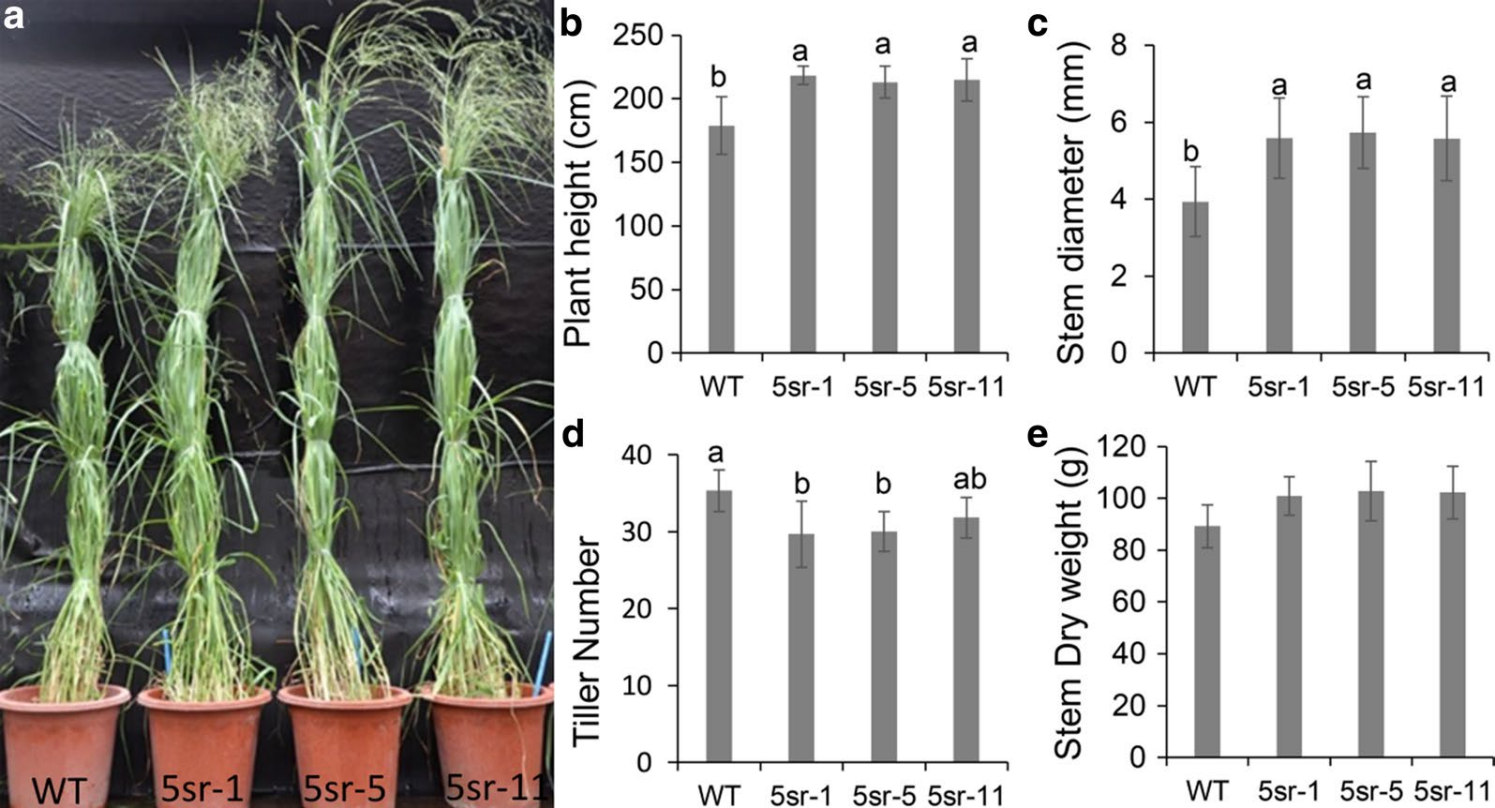
Morphological characteristics of WT and overexpression *PvPCF5*-*SRDX* transgenic plants (5sr). **a** Typical photograph of 6-month-old WT and 5sr plants. **b** Plant height. **c** The bottom internode diameter. **d** Tiller number. **e** Stem dry weight. Data shown as the mean ± SD of four biological replications (**b**, **c** had 20 technical repeats; *N* = 4, *n *= 20). Different letters represent significant differences (*P* < 0.05)

To gain insight into the molecular mechanism of how miR319 increased biomass yield and cell wall conversion in switchgrass, the activity of the miR319 target gene *PvPCF5* was suppressed by overexpression of a *PvPCF5* chimeric repressor (*PvPCF5*-*SRDX*) (Additional file 2: Data S1). The relative expression level of *PvPCF5* in the *PvPCF5*-*SRDX* transgenic lines (5sr) ranked as 5sr-5 > 5sr-1 > 5sr-11, while there was no statistical difference existed between 5sr-1 and 5sr-11 plant [34]. The 5-sr plants had significantly taller and thicker stems, and wider and longer leaves compared to WT plants (Fig. 4a–c; Additional file 4: Table S2). The stem length of 5sr plants was significantly longer (about 10 cm) than that of WT, but the tiller number was significantly reduced, which resulted in a lack of significant differences in dry weight between 5sr and WT plants (Fig. 4d, e; Additional file 4: Table S2).

**Fig. 5:**
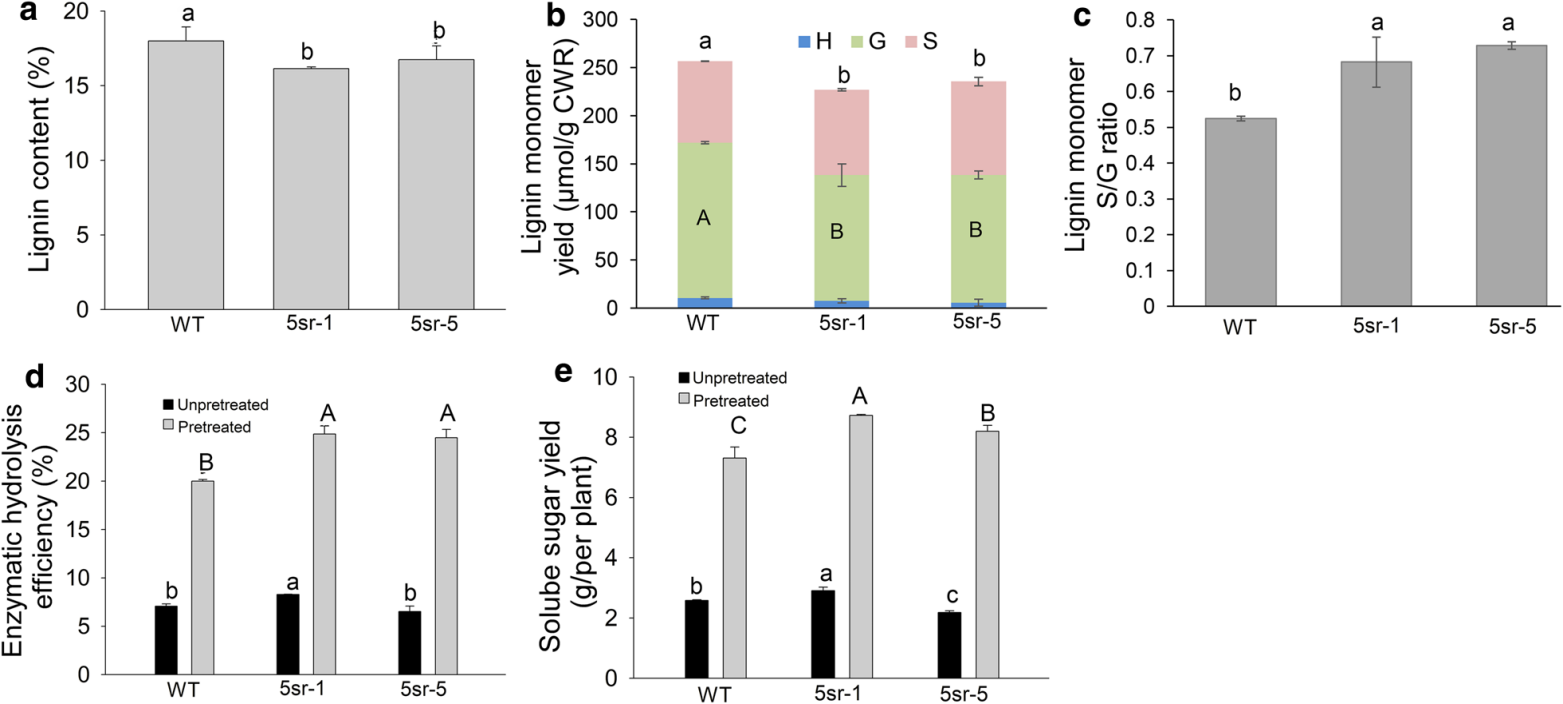
Effects of *PvPCF5*-*SRDX* overexpression on lignin content and cell wall conversion. **a** Analysis of Klason lignin content of dry stem material. **b** Comparison of lignin monomers syringyl (S), guaiacyl (G), *p*-hydroxyphenyl (H) content. **c** Comparison of lignin monomer S/G ratio. **d** Enzymatic hydrolysis efficiency analysis. **e** Analysis of soluble sugar yield of per plant. Data are shown as mean ± SD (in **b** and **c**, *N* = 2; in a, d and e had three biological repeats and five technical duplicates; *N* = 2; *n* = 5). Different letters represent significant differences (*P* < 0.05)

We also compared the lignin content of WT and 5sr plants by histochemical stain with phloroglucinol–HCl. The hand-cut stem sections of the 1NE3 of 5sr lines showed an obvious lighter red color in collenchyma and in the DMS powder compared to that of WT plant (Additional file 5: Fig. S2). Based on the plant morphology and *PvPCF5*-*SRDX* expression level, we selected two representative lines (5sr-1 and 5sr-5) for further study. Quantitative determination analysis showed the Klason lignin content of 5sr lines was significantly reduced, compared to WT plants (Fig. 5a). The total lignin monomer content of 5sr-1 and 5sr-5 was significantly lower than that of WT, which mainly due to the significantly reduced guaiacyl (G) units content in 5sr plants (Fig. 5b), which resulted in a significantly higher S/G ratio in 5sr plants than that of WT (Fig. 5c). The results indicated that suppression of *PvPCF5* reduced switchgrass lignin content mainly by reducing G unit content, which resulted in a higher S/G ratio in 5sr plants compared to WT plant.

### Overexpression of *PvPCF5*-*SRDX* improved lignocellulose conversion to sugar

We evaluated the effects of the alteration of lignin content and composition in two representative 5sr lines (5sr-1 and 5sr-5) on the conversion of lignocellulose and total sugar yield of transgenic plants. The results showed that without pretreatment the highest efficiency of enzymatic hydrolysis was observed in CWR of 5sr-1 line (8.3%). After alkali pretreatment, sugar release efficiency of 5sr-1 and 5sr-2 was 24.9% and 24.5%, which were significantly higher than that of WT (about 20%) (Fig. 5d). Calculating the total sugar yield of per plant shows alkali pretreatment significantly increased sugar yield in WT and 5sr plants. The CWR of 5sr-1 and 5sr-5 plant produced more sugar than WT after alkali pretreatment (Fig. 5e). The results demonstrated that suppression of miR319 target gene *PvPCF5* could enhance lignocellulose conversion to sugar.

### MiR319–*PvPCF5* module affected lignin compositions and chemical structure

**Fig. 6:**
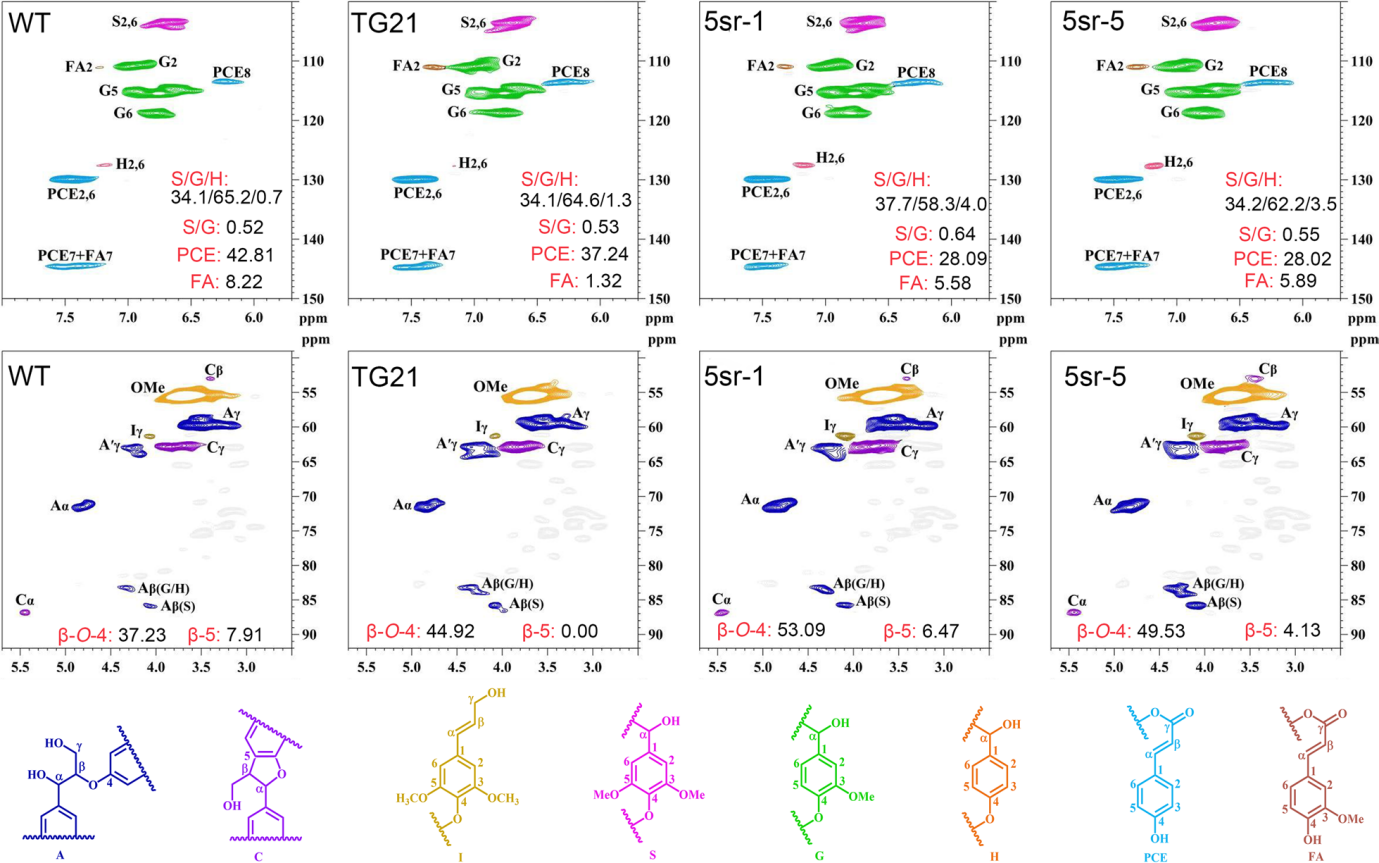
The side-chain and aromatic region in 2D-HSQC NMR spectra of WT, TG21, 5sr-1 and 5sr-5. S, syringyl; G, guaiacyl; H, *p*-hydroxyphenyl; FA, ferulate; PCE, *p*-coumaric acid ethyl ester; A, β-*O*-4 aryl ethers; C, phenylcoumarans; I, *p*-hydroxycinnamyl alcohol end-groups

To find out the effects of miR319–*PvPCF5* module on lignin compositions and chemical structure of lignin polymers, we analyzed the aromatic (δC/δH 100–150/5.7–8.0) and side-chain (δC/δH 49–92/2.5–5.7) regions of the double enzymatic lignin (DEL) by two-dimensional heteronuclear single-quantum coherence (2D-HSQC) NMR technique [35]. As shown in Fig. 6, all the tested plants exhibited similar spectral patterns. The OE-miR319 or repression of *PvPCF5* (5sr) plants had lower G units yield and higher H unit yield than that of WT, which resulted in higher S/G ratio in OE-miR319 line TG21, *PvPCF5*-SRDX lines 5sr-1 and 5sr-5. The content of *p*-coumaric acid ethyl ester (PCE) and ferulate (FA) of WT was higher than that in transgenic plants. In the 2D-HSQC spectra side-chain regions of lignin samples from different switchgrass lines, the conspicuous methoxyl groups (OMe) and the substructures, such as β-*O*-4 aryl ethers (A), phenylcoumarans (C), and *p*-hydroxycinnamyl alcohol end-groups (I), could be assigned following previous publications [35, 36]. The relative abundance of linkages was also calculated [35]. The results showed that OE-miR319 or 5sr plants had a higher content of β-*O*-4 linkage compared to that in WT. The results indicated that miR319 could, through its target gene *PvPCF5*, modulate lignin content, composition and side-chain, such as β-*O*-4 linkage content.

### Expression analyses of cell wall biosynthesis-related genes

**Fig. 7:**
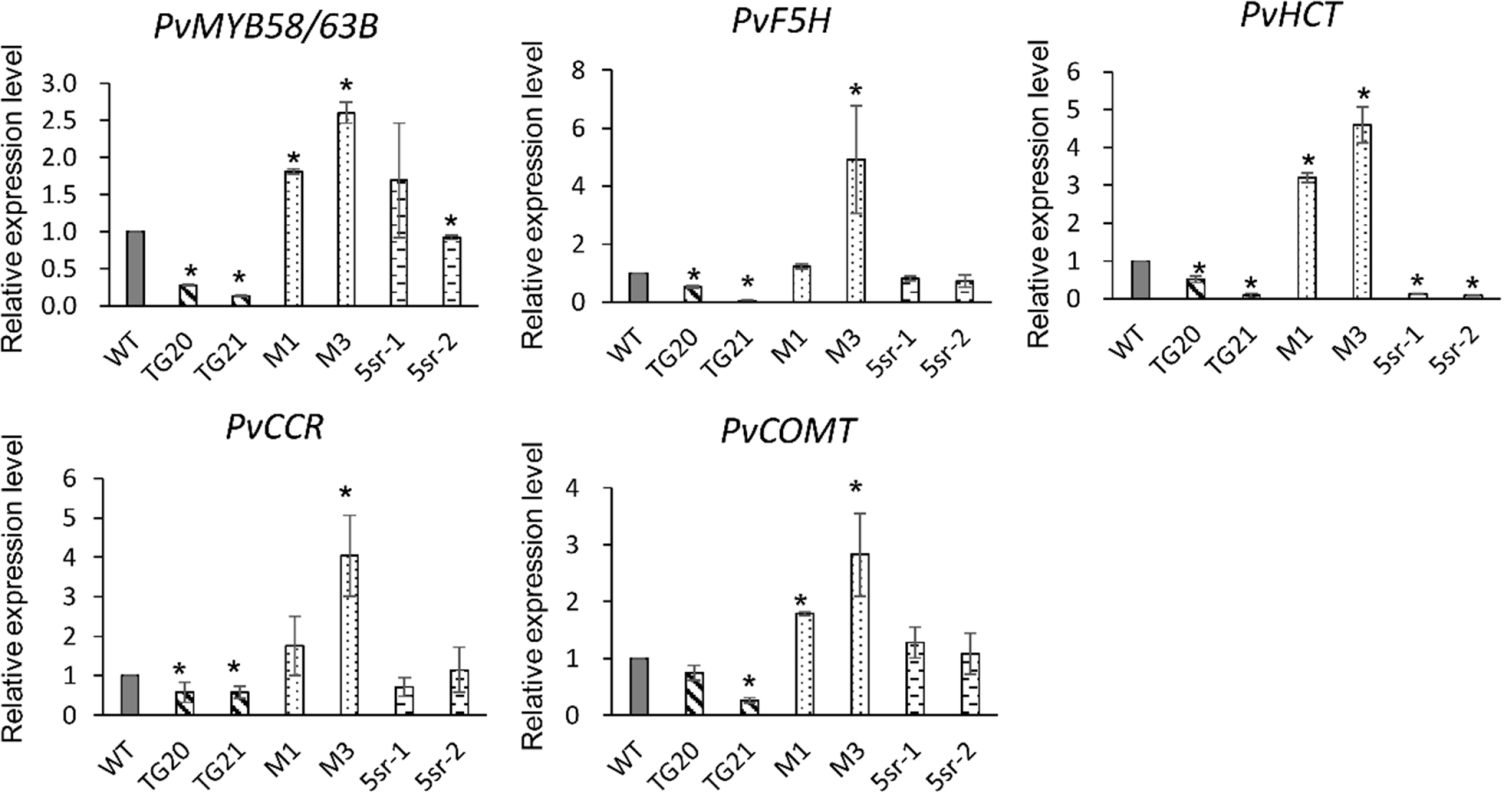
Relative expression levels of cell wall synthesis related genes in WT and transgenic plants

To explore the potential molecular mechanisms of the miR319–*PvPCF5* module in regulating switchgrass lignin content and composition, we downloaded the 2.0 Kb promoter sequences of the verified cell wall biosynthesis-related TFs and genes in switchgrass from phytozome (https://phytozome.jgi.doe.gov/pz/portal.html). Then, predicted TCP binding sites (A/T/GGGACCAC) using JASPAR software (relative score > 0.9) with AtTCP4 as model (Additional file 6: Table S3). Three genes (*PvMYB58/63*, *PvF5H* (*ferulate 5*-*hydroxylase*) and *PvHCT* (*shikimate hydroxycinnamoyltransferase*)) that have a TCP binding site were selected to examine their expression pattern by qRT-PCR in WT, OE-miR319, *MIM319*, and 5sr lines. All the three tested genes showed lower expression in OE-miR319 plants, and also in 5sr lines except *PvF5H* compared to WT plants (Fig. 7). Two lignin synthesis genes, *PvCCR* (*cinnamoyl CoA reductase*) and *PvCOMT* (*caffeic acid O*-*methyltransferase*), showed a similar expression pattern as *PvPCF5* [11], showed reduced expression in OE-miR319 compared to WT plants, but had no expression difference in 5sr plants compared to WT plants. The results indicate the reduced lignin content in OE-miR319 and 5sr plants might be largely due to regulating the expression of *PvMYB58/63* and *PvHCT.*

## Discussion

Stem characters and degree of lignification are crucial traits for aboveground biomass yield and feedstock quality of perennial biofuel plants. Enhancing tiller density, height and/or reducing stem lignin content by biotechnology methods could effectively improve switchgrass biofuel characteristics [21, 37]. MiR319–*TCP* module has been demonstrated to play a key role in plant development, such as promoting cell proliferation during leaf organ morphogenesis [22–26], delaying leaf senescence [27] and affecting flower development [28]. However, little is known about the biological function of miR319–*TCP* module in regulating stem development of perennial biofuel grasses, such as switchgrass.

The expression level of miR319 was varied with plant development [28]. In this study, we found that miR319 expression level was gradually reduced during switchgrass stem development. To investigate the biological function of miR319 in switchgrass stems, we produced transgenic plants with altered expression of miR319. Our data indicated miR319 kept conserved functions in switchgrass as in other plant species in improving leaf width and thickness, suppressing tiller numbers, and enlarging stem diameters [25, 26]. In addition, miRNAs also showed species-specific functions, such as the opposite effect of miR393 in regulating tiller number among different plant species [20, 38, 39]. In this work, we observed miR319 played a positive role in improving stem elongation. However, overexpression of miR319 in creeping bentgrass plants resulted in similar plant height as the wild type [25]. In rice, miR319 has been reported as a miRNA that suppresses plant height [26]. Therefore, the effect of miR319 on stem elongation could be different in different plant. We had found OE-miR319 promoted ethylene biosynthesis in switchgrass, and reduced sensitivity of transgenic plants to exogenous ethylene [34]. Reduced ethylene sensitivity has been reported to promote growth in a wide variety of plants [40], and may at least partially be, contributing to longer stems of OE-miR319 switchgrass lines.

Previous reports indicate miR319 regulate plant development through posttranscriptional regulation of the class II TCPs mRNA abundance [22–29]. In our earlier report, mRNA of five tested *PvTCPs* (*PvPCF5*, *6*, *7*, *8* and *PvTCP21*) presumed as miR319–target genes was significantly down-regulated in OE-miR319 plants [34]. In this study, *PvPCF7* and *PvPCF8* showed higher expression level in developed stems, and were negatively correlated with miR319 expression. Similarly, *AtTCP4*, a positive lignin content regulator, showed a similar expression pattern during *Arabidopsis* development [33]. However, the miR319 target *PvPCF5/6/21* showed the opposite expression pattern with *PvPCF7/8* during stem development. Here, we verified that transgenic manipulation of miR319 or its target gene *PvPCF5* influence switchgrass stem growth and lignification, while their contribution to endogenous regulatory networks for these processes still remains unclear.

Stem development and senescence always accompanied with lignin deposition. Lignin is the main repressor in conversion of lignocellulose feedstock [16]. Two sequential processes of stem lignification coordinated by miR319–TCPs module negatively control biosynthesis of JA [27]. Exogenous application of JA promotes plant transition from vegetative phase to floral phase [41], and mediates cell wall lignin accumulation [42]. Our data clearly showed that miR319–*PvPCF5* pathway negatively regulated stem lignin content. Similar results were also reported in *Arabidopsis* [33]. However, we did not find differences in flowering time between WT and transgenic plants.

Lignin as one of the most abundant natural biopolymer, is composed with S, G and H units, its linkage mainly by aryl-ether (such as β-*O*-4) and carbon–carbon (β-β, β-5, etc.) bonds [43]. Theoretically, higher S unit content and/or S/G ratio always accompanied with a higher proportion of β-*O*-4 linkages [35, 44], which will be benefit to subsequent depolymerization and upgrading [45]. In this study, we found miR319–*PvPCF5* module increased S/G ratio and β-*O*-4 linkage of switchgrass. A small amount of β-β linkages was also be found in side-chain regions of switchgrass lignin [46]. However, we did not detect any β-β bond in tested DELs, which may be due to different lignin extraction and detection methods [35, 46].

NAC-MYB-based transcriptional regulatory system regulates secondary cell wall biosynthesis and has been well studied in switchgrass [11, 13, 47, 48]. In *Arabidopsis*, miR319 target gene *TCP4* could directly activate expression of NAC TF *AtVND7* (one master regulator in cell wall biosynthesis), which resulted in increases in lignin and cellulose content [33]. However, no TCP binding site was predicted in the promoter of homolog gene *PvVND7* using JASPAR software (Additional file 6: Table S3). In this study, we found the promoter of *PvMYB58/63B* (an activator of lignin biosynthesis [33]) has a predicted TCP binding site, and *PvMYB58/63B* was significantly down-regulated in the transgenic plants of OE-miR319 or 5sr, which indicated that *PvMYB58/63B* might be a direct target of *PvPCF5* in switchgrass. In switchgrass, overexpression of *PvMYB58/63* significantly increased lignin content by elevating lignin biosynthesis genes [33]. However, lignin content showed no obvious effects in down-regulated *PvMYB58/63* plants in rice [49] or switchgrass [33], which indicates other pathways may be involved in miR319–*PvPCF5* reducing lignin content. In this study, we also found the promoters of *PvHCT* and *PvF5H* had predicted TCP binding sites (Additional file 6: Table S3). Furthermore, two lignin biosynthesis-related genes, *PvCCR* and *PvCOMT* showed a similar expression pattern as *PvPCF5* [33]. The four genes were all down-regulated in OE-miR319 plants, but only *PvHCT*, a repressor for lignin content and plant height [10, 50], was significantly down-regulated in 5sr lines. The results indicate that miR319–*PvPCF5* mediated a multiplex and complex pathway to negatively regulate lignin content, and *PvMYB58/63B* and *PvHCT* could be considered as candidate targets of *PvPCF5* in further studies.

Biomass yield and feedstock quality, as two of the major factors that affect biofuel production, always show a tight trade-off. Increasing biomass yield is also accompanied by a higher lignin level, such as ectopic expression *ZmGA20ox* switchgrass plants [51]. Reducing lignin content by directly down-regulating the lignin biosynthesis enzyme or cell wall-related TFs also resulted in a stunted phenotype [11–13]. Fortunately, the negative effect on plant growth may be avoided by regulating some transcription factors that has no direct association with the cell wall biosynthesis. For example, overexpression of *PvERF001* in switchgrass improved biomass yield and enzymatic saccharification [14]. Suppressing miR156-target *PvSPL2* activity effectively improved switchgrass biomass yield and lignocellulose conversion [52]. In this study, we show miR319–*PvPCF5* regulatory pathway is another option for simultaneously improving biomass yield and enzymatic efficiency.

## Conclusions

Overexpression of miR319 or repressing its target gene *PvPCF5* promoted switchgrass stem elongation, reduced lignin content and increased S/G ratio and β-*O*-4 linkages, which resulted in improving biomass yield and enzymatic hydrolysis efficiency, and elevating sugar release of per plant. Furthermore, we showed secondary cell wall biosynthesis TF *PvMYB58/36* and lignin biosynthesis gene *PvHCT* were significantly down-regulated by miR319–*PvPCF5* module. This is the first report on the effects of miR319–*PvPCFs* pathway in improving plant characteristics for biofuel production in a C_4_ plant. The finding could also be transferable to other grasses for forage quality improvement through genetic manipulation.

## Materials and methods

### Plant materials

Switchgrass cultivar Alamo was used in this study. Generation of the switchgrass transgenic plants was reported in our previous paper [34]. The transgene and the selectable marker gene *hpt* were driven by the CaMV 35S promoter in the T-DNA region of vector pZH01, respectively. The vectors were introduced into *A. tumefaciens* strain EHA105, separately, for plant transformation. More data were presented in Additional file 2: Data S1.

### Plant phenotype analysis

Each tested line had four plants propagated by split-tillers planted in pots and maintained in a greenhouse (16 h light/8 h dark) for 6 months (as four biological repeats). Twenty R3 stage tillers from each plant were used to measure the phenotypes (as one technical repeats). Leaf length of the second leaf from top of R3 stage tillers was measured using a tape and the leaf blade width and thickness 1 cm from the base of each leaf were determined using vernier calipers. The bottom internode diameter (at aboveground 5 cm) of R3 stage tillers were measured using vernier calipers. Internode number, internode length, rachis length, and stem length were measured from R3 stage tillers using a tape. The aboveground biomass of each plant was harvested, separated into leaves and stems, and dried at 65 °C for 48 h, then weighted separately.

### Determination of Klason lignin content and enzymatic hydrolysis efficiency

Twenty R3 stage stems of each plant were ground and filtered through a 1 mm sieve for the following study. The DMS powder was used to extract cell wall residues (CWR) as described by Chen and Dixon [53]. The Klason methods described by Jung et al. [54] were used to quantify the lignin content of the DMS. A two-stage acid hydrolysis method was used to determine the total carbohydrate content of CWR [54]. In brief: CWR was stored with 72% sulfuric acid for 1 h and then dilute sulfuric to 4%, then kept at 121 °C for 1 h. The supernatant liquid was used to test the total carbohydrate using an HPLC system equipped with a Hi-Plex Ca column (7.7 × 300 mm, Agilent Technology, USA), LC-20AT pump (Shimadzu, Japan) and RID-10A refractive index detector (Shimadzu, Japan) [55]. For enzymatic hydrolysis assay, DMS was digested directly by exposure to 0.5 ml enzyme mixture (4.0 g/l, Imperial Jade Biotechnology Co., Ltd) or pretreated with 0.25 M NaOH and then treated with the same amount enzyme by following the procedure mentioned our previous report [56]. The enzymatic hydrolysis efficiency is the percentage of released carbohydrate content after enzymatic hydrolysis to the total carbohydrate content. The soluble sugar yield of per plant was calculated using total carbohydrate content of each plant and enzymatic hydrolysis efficiency.

### Lignin monomer content determination

The lignin monomers were identified and quantified by GC–MS analysis using a gas chromatograph (Hewlett-Packard 5890 series II) with a series of mass selective detector (5971) according to the thioacidolysis method [57]. Two biological duplicates of each line were performed.

### Histological analysis

To assess lignin content, we added 0.15 g DMS samples to 2 ml tubes and stained with 2 ml phloroglucinol/HCl (1/12, w/w) for 5 min. The hand-cut section of the middle of the first internode from top of E3 stage tiller was stained with phloroglucinol/HCl for 1 min. Photos were taken with a digital camera on a microscope (Nikon, C-DSS230).

### 2D-HSQC NMR sample preparation and NMR analysis

For NMR analysis, 5.0 g DMS samples were placed into 50 ml cobalt oxide tanks, and ground to fine powder with 18 cobalt oxide balls (1 cm in diameter) at a speed of 600 rpm. To prevent the oxidation of samples in the ball-milling process, the interval of 10 min for each grinding was set at 5 min, and the total ball-milling time was 6 h. An appropriate amount of ball-milled sample was taken and placed in 20 mM cellulose complex enzyme solution (pH 4.8) for enzymatic hydrolysis at 50 °C for 48 h, and repeated. Samples were collected by centrifuging after enzyme treatment, washed with distilled water 3 times, and freeze-dried, as the double enzymatic lignins (DELs) [35]. The dried samples were dissolved in DMSO as lignin samples. Bruker standard pulse program was used to detect lignin monomer structure with Bruker Avance-III 400 MHz NMR instrument [35].

### Quantitative real-time PCR analysis

The stem RNA was extracted using Trizol regent. One μg RNA was used to reverse transcription into the first strand of cDNA (Takara RR047 kit) for qRT-PCR analysis using the primers list in Additional file 7: Table S4.

### Statistical analysis

In each experiment, all leaf and stem characteristics were collected from four biological replicates (*N* = 4) with 20 technical repeats (*n* = 20); tiller number and dry weight data were assessed from four biological replicates (*N* = 4); lignin content and enzymatic hydrolysis efficiency were collected from three biological replicates (*N* = 3) with five technical repeats (*n* = 5). Data from biological replicates were used for statistical analysis by one-way *ANOVA*. Treatments were compared by Duncan’s multiple range test (*P* < 0.05). PROC GLM for ANOVA in SAS 8.2 (SAS Institute, Cary, NC, USA) was used for analyses.

## Supplementary information

**Additional file 1: Fig. S1.** mRNA amount of miR319 and target *PvPCFs* in different part of the second internode from top of R1 stage stems. (**a**) the second internode of R1 stage tiller was cut into 18 segments (about 1 cm long each) and phloroglucinol staining was performed. The expression level of miR319 (**b**) and *PvPCFs* (**c**) in the first, sixth, twentieth and eighteenth segment.

**Additional file 2: Data S1.** Supplementary methods, generation of agrobacterium-mediated transgenic switchgrass plants.

**Additional file 3: Table S1.** Chemical composition of WT and miR319 expression altered transgenic plants.

**Additional file 4: Table S2.** Morphological characteristics of WT and 5sr plants.

**Additional file 5: Fig. S2.** Typical photograph of Phloroglucinol-HCl staining assay of lignin content in the middle of the first internode of E3 stage cross-sections of WT and 5sr (a) and in the dry materials powder of stems (b).

**Additional file 6: Table S3.** TCP binding sites were predicted in the promoter region of cell wall synthesis-associated transcription factors and genes using JASPAR software (relative score > 0.9).

**Additional file 7: Table S4.** The primers used for lignin synthesis related genes qRT-PCR tests.

## Data Availability

All data generated or analyzed in the present study are included in this article and in additional information.

## Abbreviations

miRNA: MicroRNA
miR319: MicroRNA319
*MIM319*: A target mimicry of miR319
OE-miR319: Overexpression of miR319
TFs: Transcription factors
AP2/ERF: APETALA2/ethylene responsive factor
NAC: NAM/ATAF/CUC
MYB: MYELOBLASTOSIS
TCP: TEOSINTE BRANCHED1, CYCLODEA, PROLIFERATING FACTORS/PCF
ICK1: Cyclin-dependent kinase inhibitor 1
BR: Brassinolide
JA: Jasmonic acid
DWARF4: BR biosynthesis key enzyme
LOX2: Lipoxygenase 2
DMS: Dry materials of stems
VND7: VASCULAR-related NAC-DOMAIN7
ERF: Ethylene response factor
1NE3: The first internode from the top of the E3 stage tiller
qRT-PCR: Quantitative reverse transcription polymerase chain reaction
5sr: PvPCF5-SRDX transgenic lines
VT: Vascular tissues
C: Collenchyma
CWR: Cell wall residues
WT: Wild type
TG: Transgenic
4CL1: 4-Coumarate CoA ligase 1
CCR: Cinnamoyl CoA reductase
F5H: Ferulate 5-hydroxylase
COMT: Caffeic acid *O*-methyltransferase
HCT: Shikimate hydroxycinnamoyltransferase
CAD: Cinnamyl alcohol dehydrogenase
S: Syringyl
G: Guaiacyl
H: p-Hydroxyphenyl
FA: Ferulate
PCE: *p*-Coumaric acid ethyl ester
DELs: Double enzymatic lignins
2D-HSQC: Two-dimensional heteronuclear single-quantum coherence
NMR: Nuclear magnetic resonance
