# Supplementary material for: Heteroexpression of Osa-miR319b improved switchgrass biomass yield and feedstock quality by repression of PvPCF5
Source: Biotechnol Biofuels. 2020 Mar 19;13:56. doi: 10.1186/s13068-020-01693-0 (PMC7081615; doi:10.1186/s13068-020-01693-0)
Supplement: Supplementary file 1 — Additional file 1: Fig. S1. mRNA amount of miR319 and target PvPCFs in different part of the second internode from top of R1 stage stems. (a) the second internode of R1 stage tiller was cut into 18 segments (about 1 cm long each) and phloroglucinol staining was performed. The expression level of miR319 (b) and PvPCFs (c) in the first, sixth, twentieth and eighteenth segment. [file 13068_2020_1693_MOESM1_ESM.docx]

**Additional file 1**

**
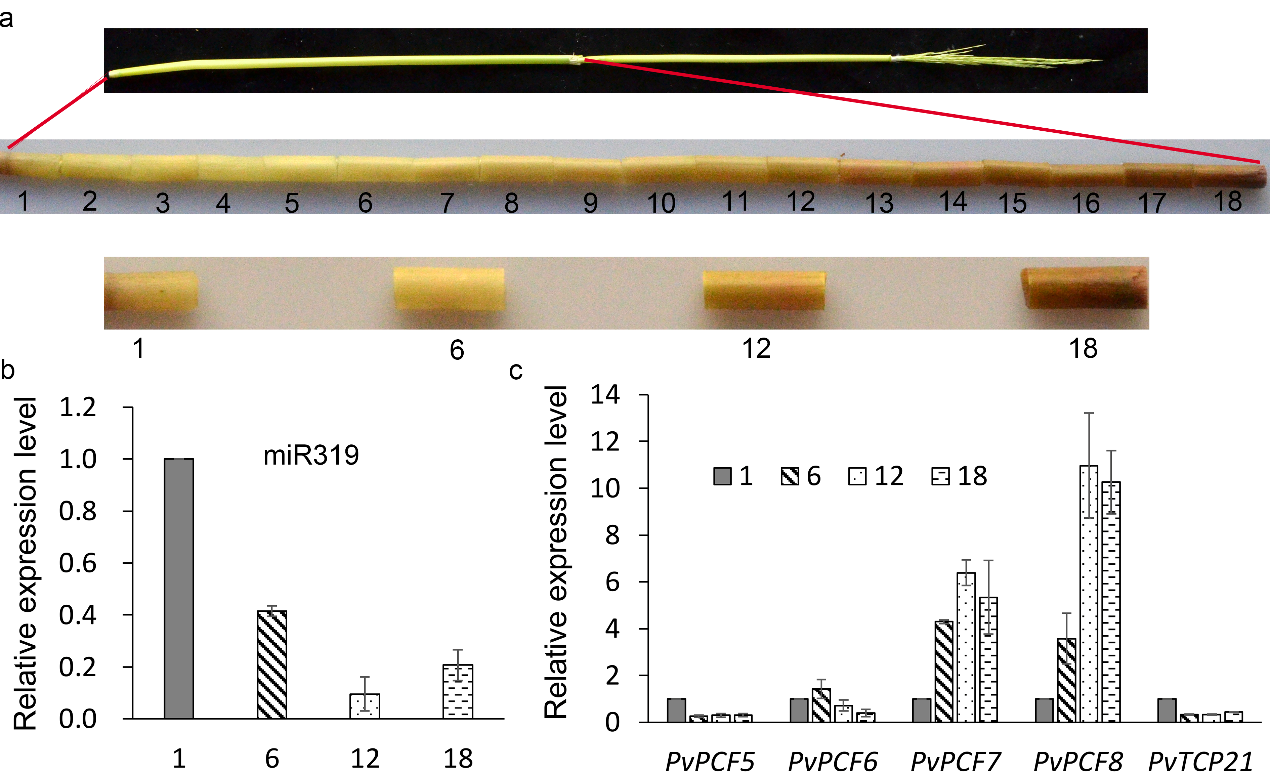
**

Fig. S1 mRNA amount of miR319 and target *PvPCFs* in different part of the second internode from top of R1 stage stems. **a** The second internode of R1 stage tiller was cut into 18 segments (about 1cm long each) and phloroglucinol staining was performed. The expression level of miR319 (**b**) and *PvPCFs* (**c**) in the first, sixth, twentieth and eighteenth segment.
