# Supplementary material for: Heteroexpression of Osa-miR319b improved switchgrass biomass yield and feedstock quality by repression of PvPCF5
Source: Biotechnol Biofuels. 2020 Mar 19;13:56. doi: 10.1186/s13068-020-01693-0 (PMC7081615; doi:10.1186/s13068-020-01693-0)
Supplement: Supplementary file 2 — Additional file 2: Data S1. Supplementary methods, generation of agrobacterium-mediated transgenic switchgrass plants. [file 13068_2020_1693_MOESM2_ESM.docx]

**Additional file 2：Data S1**

**Supplementary methods, Generation of Agrobacterium-mediated transgenic switchgrass plants**

Generation of *Agrobacterium-*mediated transgenic switchgrass was reported by Liu et al. (2019). Briefly, the full length of rice *Osa-MIR319b* cDNA (AK241923) was retrieved from NCBI and cloned into the binary vector pZH01. An artificial target mimicry against miR319 was engineered from the Arabidopsis *IPS1* gene (AF236376.1) as previous reported and cloned to binary vector pZH01 [1, 2]. To suppress the miR319 target gene *PvPCF5* (Pavir.J362100.1), the full length cDNA sequence was retrieved from switchgrass genome database [3] and an artificial gene of *PvPCF5-SRDX* was cloned to pZH01[4]. The transformation procedure following our previous report [5].

DNA of regenerated plants was extracted using the CTAB method and was used for PCR tests. Total RNA of the flag leaves was extracted with Trizol reagent and then used for synthesis of the first strand cDNA according to the introduction of Takara RR047A kit. For measuring the mature miR319 expression level, the putative miR319 stem-loop primer was used for synthesis the first strand cDNA. The SYBR green supermix (Takara RR420) was used for quantitative real-time (qRT) PCR analysis using the EcoTM Real-Time PCR System (Illumina, EC-100-1001, CA, USA).

**Supplementary results**

**Molecular testing of transgenic plants** was reported by Liu et al. [34] and is briefly described here. All generation plants were first verified by PCR amplification. PCR positive lines were then used to determine the mature miR319 level or *PvPCF5* content by qRT-PCR. Three overexpression *Osa-MIR319b* lines (TG21, TG20 and TG1), holding significantly higher miR319 content (202-, 172- and 41- fold) compared with WT, were selected for further study. The miR319 level in M1, M3 and M4 lines only showed 0.16-, 0.10- and 0.26-fold of that in WT. The *PvPCP5* expression level of three *PvPCF5* loss-function (overexpression *PvPCF5-SRDX*) lines (5sr-1, 5sr-5 ad 5sr-11) upregulated to 2.7- , 3.6- and 2.3-fold than WT. Those lines were then selected for this study.

**Reference**

1. Franco-Zorrilla JM, Valli A, Todesco M, Mateos I, Puga MI, Rubio-Somoza I, Leyva A, Weigel D, Garcia JA, Paz-Ares J. Target mimicry provides a new mechanism for regulation of microRNA activity. Nat Genet. 2007;39:1033-7.
2. Todesco M, Rubio-Somoza I, Paz-Ares J, Weigel D. A collection of target mimics for comprehensive analysis of MicroRNA function in *Arabidopsis thaliana*. PLoS Genet. 2010;6:e1001031.
3. Xie Q, Liu X, Zhang Y, Tang J, Yin D, Fan B, Zhu L, Han L, Song G, Li D. 2017. Identification and characterization of microRNA319a and its putative target gene, *PvPCF5*, in the bioenergy grass switchgrass (*Panicum virgatum*). Front Plant Sci. 2017;8:396.
4. Cen HF, Ye WX, Liu YR, Li DY, Wang KX, Zhang WJ. Overexpression of a chimeric gene, *OsDST-SRDX*, improved salt tolerance of perennial ryegrass. Sci Rep. 2016;6:27320.
5. Liu YR, Cen HF, Yan JP, Zhang YW, Zhang WJ. Inside out: high-efficiency plant regeneration and *Agrobacterium*-mediated transformation of upland and lowland switchgrass cultivars. Plant Cell Rep. 2015;34:1099–108.
