# Supplementary material for: Heteroexpression of Osa-miR319b improved switchgrass biomass yield and feedstock quality by repression of PvPCF5
Source: Biotechnol Biofuels. 2020 Mar 19;13:56. doi: 10.1186/s13068-020-01693-0 (PMC7081615; doi:10.1186/s13068-020-01693-0)
Supplement: Supplementary file 3 — Additional file 3: Table S1. Chemical composition of WT and miR319 expression altered transgenic plants. [file 13068_2020_1693_MOESM3_ESM.docx]

**Additional file 3**

Table S1 Chemical composition of WT and miR319 expression altered transgenic plants.

| Lines | Glucan (mg/g CWR) | Xylan (mg/g CWR) | Arabinan (mg/g CWR) | Xylitol (mg/g CWR) | Total carbohydrate (mg/g CWR) |
| --- | --- | --- | --- | --- | --- |
| WT | 321.51± 1.78 | 96.62± 1.51 | 12.24± 0.86 | 6.54± 0.42 | 436.90± 2.01 |
| TG21 | 340.44± 6.18 | 92.05± 6.69 | 13.41± 0.10 | 5.76± 0.28 | 451.66± 13.05 |
| TG20 | 309.52± 24.62 | 83.53± 1.43 | 13.57± 0.45 | 6.71± 0.97 | 413.33± 27.48 |
| TG1 | 328.45± 18.71 | 93.47± 4.34 | 13.31± 0.56 | 6.58± 0.17 | 441.81± 23.78 |
| M4 | 323.31± 2.46 | 87.19± 1.29 | 13.18± 0.85 | 6.59± 0.43 | 430.28± 0.11 |
| M1 | 327.96± 4.37 | 97.59± 1.54 | 14.54± 0.39 | 5.58± 0.09 | 445.67± 3.13 |
| M3 | 340.64± 15.81 | 92.98± 12.27 | 14.87± 1.42 | 5.36± 0.03 | 453.85± 29.53 |

The data shown as the mean of three biological replications (with five technical repeats) ± SD. There were no statistical significant differences determined by Duncan’s multiple range test (*P* < 0.05).
