# Supplementary material for: Heteroexpression of Osa-miR319b improved switchgrass biomass yield and feedstock quality by repression of PvPCF5
Source: Biotechnol Biofuels. 2020 Mar 19;13:56. doi: 10.1186/s13068-020-01693-0 (PMC7081615; doi:10.1186/s13068-020-01693-0)
Supplement: Supplementary file 4 — Additional file 4: Table S2. Morphological characteristics of WT and 5sr plants. [file 13068_2020_1693_MOESM4_ESM.docx]

**Additional file 4**

Table S2 Morphological characteristics of WT and 5sr plants

| Line | Leaf length (cm) | Stem length (cm) | Blade width (mm) | Richis length (cm) | Internode length (cm) |
| --- | --- | --- | --- | --- | --- |
| WT | 51.57±3.77 b | 110.21±18.18 c | 12.00±2.30 b | 68.62 ±12.46 c | 17.51 ±2.06 b |
| 5sr-1 | 62.32±6.29 a | 127.43±14.85 ab | 13.85±1.03 ab | 85.57 ±8.06 a | 20.24 ±1.40 a |
| 5sr-5 | 59.64±5.95 a | 133.05±14.67 b | 15.38±1.15 a | 81.85 ±7.16 ab | 21.98 ±2.92 a |
| 5sr-11 | 48.85±2.20 b | 139.65±6.84 a | 15.69±0.99 a | 78.74±4.24 b | 20.50 ±1.65 a |

The data shown as the mean of four biological replicates (with twenty technical repeats) ± SD. The different letters indicted statistical significant differences determined by Duncan’s multiple range test (*P* < 0.05).
