# Supplementary material for: Heteroexpression of Osa-miR319b improved switchgrass biomass yield and feedstock quality by repression of PvPCF5
Source: Biotechnol Biofuels. 2020 Mar 19;13:56. doi: 10.1186/s13068-020-01693-0 (PMC7081615; doi:10.1186/s13068-020-01693-0)
Supplement: Supplementary file 5 — Additional file 5: Fig. S2. Typical photograph of Phloroglucinol-HCl staining assay of lignin content in the middle of the first internode of E3 stage cross-sections of WT and 5sr (a) and in the dry materials powder of stems (b). [file 13068_2020_1693_MOESM5_ESM.docx]

**Additional file 5**

**
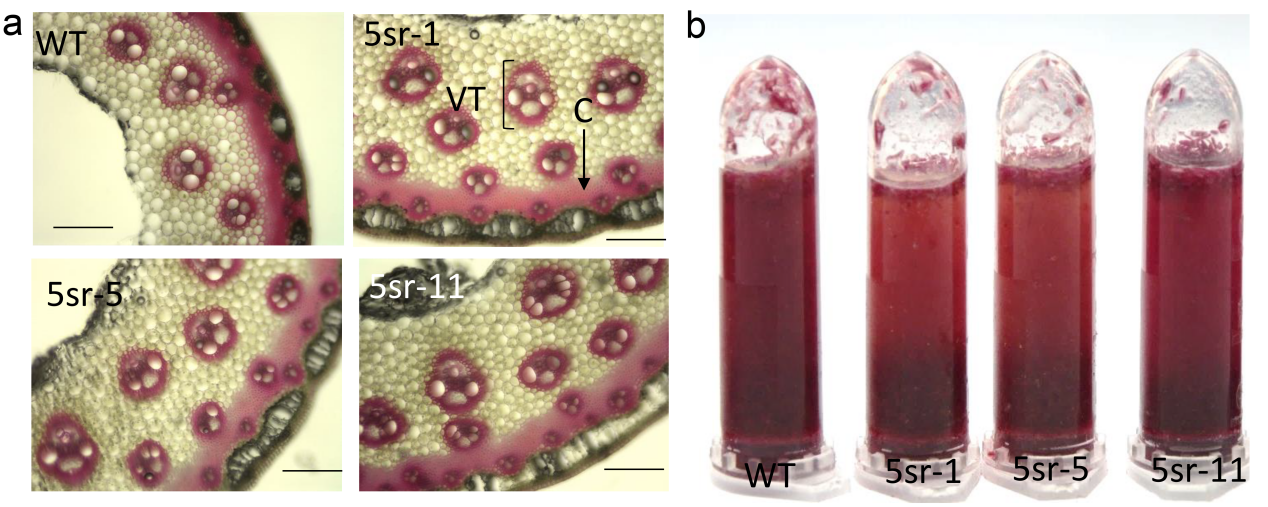
**

Fig. S2 Typical photograph of Phloroglucinol-HCl staining assay of lignin content in the middle of the first internode of E3 stage cross-sections of WT and 5sr (a) and in the dry materials powder of stems (b). C, collenchyma; VT, vascular tissue. Scale bars indicate 5 μm.
