# Supplementary material for: Heteroexpression of Osa-miR319b improved switchgrass biomass yield and feedstock quality by repression of PvPCF5
Source: Biotechnol Biofuels. 2020 Mar 19;13:56. doi: 10.1186/s13068-020-01693-0 (PMC7081615; doi:10.1186/s13068-020-01693-0)
Supplement: Supplementary file 6 — Additional file 6: Table S3. TCP binding sites were predicted in the promoter region of cell wall synthesis-associated transcription factors and genes using JASPAR software (relative score > 0.9). [file 13068_2020_1693_MOESM6_ESM.docx]

**Additional file 6**

Table S3 TCP binding sites were predicted in promoter of cell wall synthesis-associated transcription factors and genes using JASPAR software (relative score >0.9)

| Define | Gene ID | Model name | Score | Relative score (>0.9) | Start | End | Strand | predicted site sequence |
| --- | --- | --- | --- | --- | --- | --- | --- | --- |
| PvSND7 | Pavir.J20890 | AtTCP4 | - | - | - | - | - | - |
| PvMYB58/63B | Pavir.Ga00752 | AtTCP4 | 12.37 | 0.95 | -1171 | -1178 | 1 | TGGACCAC |
| PvMYB58/63A | Pavir.Gb00587 | AtTCP4 | - | - | - | - | - | - |
| PvMYB42/85 | Pavir.Bb02654 | AtTCP4 | - | - | - | - | - | - |
| PvSND2A | Pavir.Eb02718 | AtTCP4 | - | - | - | - | - | - |
| PvSWN2 | Pavir.J05241 | AtTCP4 | - | - | - | - | - | - |
| PvHCT | Pavir.J37079 | AtTCP4 | 10.108 | 0.91 | -1267 | -1274 | -1 | AGGCCCAC |
| PvHCT | Pavir.J37079 | AtTCP4 | 15.077 | 1.00 | -684 | -691 | -1 | GGGACCAC |
| PvF5H | Pavir.Ia01427 | AtTCP4 | 10.108 | 0.91 | -1940 | -1947 | -1 | AGGCCCAC |
| PvF5H | Pavir.Ia01427 | AtTCP4 | 12.37 | 0.95 | -1483 | -1490 | 1 | TGGACCAC |
| PvCCR | Pavir.Fa00937 | AtTCP4 | - | - | - | - | - | - |
| PvCOMT | Pavir.J35577 | AtTCP4 | - | - | - | - | - | - |
| PvPAL | Pavir.Ab02345 | AtTCP4 | - | - | - | - | - | - |
