# Supplementary material for: Heteroexpression of Osa-miR319b improved switchgrass biomass yield and feedstock quality by repression of PvPCF5
Source: Biotechnol Biofuels. 2020 Mar 19;13:56. doi: 10.1186/s13068-020-01693-0 (PMC7081615; doi:10.1186/s13068-020-01693-0)
Supplement: Supplementary file 7 — Additional file 7: Table S4. The primers used for lignin synthesis related genes qRT-PCR tests. [file 13068_2020_1693_MOESM7_ESM.docx]

**Additional file 7**

Table S4 The primers used for lignin synthesis related genes qRT-PCR tests

| Gene ID | Forward primer | Reverse primer |
| --- | --- | --- |
| PvMYB58/63B | CTTCACAGCCGAGGAGGAGGAG | CTTCTTCAGGTGCGTGTTCCAGAC |
| PvHCT | GGCGGCTTCGTGTACGTGATG | CGGCGAACACCTTCCTGAACTC |
| PvF5H | GGCGAGGACCAGGAGGAGTTC | GGGTGCGGCGGTTGATGC |
| PvCOMT | CCTGCCGATGACGCTCAAGAAC | CCGCGCCACCACCTCCTC |
| PvCCR | GCGTCGTGGCTCGTCAA | TCGGGTCATCTGGGTTCCT |
| Ubq | CAGCGAGGGCTCAATAATTCCA | TCTGGCGGACTACAATATCCA |
| PvPCF5 | CCATCCAGTTCTACGACGTG | AGCCAGTCGACGGCCTTGCT |
| PvPCF7 | GGCAGCGTCAAGTCCTTGTTCC | TGGTGCTGGTTGGACTGGAGAG |
| PvPCF6 | GCCGAGCACAAGCAGTCCTG | CGACGTGAGAGGTGAAGTTGAAGG |
| PvPCF8 | AGCACCTCCGAGACCAGCAAG | CGAATGAGCCGCCGTTGTAGG |
| PvTCP21 | TGGGTGTCAACAGTAGGGGG | GGGCTGAACAGGAAGGGTATG |
